# Gene sequencing applications to combat oral-cavity related disorders: a systematic review with meta-analysis

**DOI:** 10.1186/s12903-023-03541-7

**Published:** 2024-01-17

**Authors:** Nishath Sayed Abdul, Mahesh Shenoy, Naveen Rami Reddy, Sunila Bukanakere Sangappa, Ganiga Channaiah Shivakumar, Marco Di Blasio, Marco Cicciù, Giuseppe Minervini

**Affiliations:** 1 Faculty of Oral Pathology, Department of OMFS and Diagnostic Sciences, Riyadh Elm University, Riyadh, Kingdom of Saudi Arabia; 2 Dept of Prosthodontics, College of Dentistry, Jazan University, Jazan, Kingdom of Saudi Arabia; 3 Department of Prosthodontics and Crown & Bridge, JSS Dental College and Hospital, JSS Academy of Higher Education and Research, Mysuru, Karnataka India; 4 Department of Oral Medicine and Radiology, People’s College of Dental Sciences and Research Centre, People’s University, Bhopal, Madhya Pradesh India; 5 Department of Medicine and Surgery, University Center of Dentistry, University of Parma, 43126 Parma, Italy; 6 Department of General Surgery and Surgical-Medical Specialties, School of Dentistry, University of Catania, 95124 Catania, Italy; 7 Saveetha Dental College & Hospitals Saveetha Institute of Medical & Technical Sciences, Saveetha University, 600 077 Chennai, India; 8 Multidisciplinary Department of Medical-Surgical and Dental Specialties, University of Campania “Luigi Vanvitelli”, Caserta, 81100 Italy

**Keywords:** Gene sequencing, Oral cavity, Disorders, DNA sequencing, Oral health

## Abstract

Gene sequencing (GS) has numerous applications in combatting oral-cavity related disorders, including identifying genetic risk factors for diseases, developing targeted therapies, and improving diagnostic methods. It can help identify specific genetic mutations or variations that increase the risk of developing oral-cavity related disorders, such as oral cancer, periodontal disease, and cleft lip and palate. By the means of the following investigation, our primary objective was to assess the impact of GS technique in diagnosing and potentially treating diseases of the oral cavity by the means of a systematic review and meta-analysis. We commenced by defining the terms "gene sequencing," "oral cavity," and "disorders" as the important elements in our investigation's subject. Next, relevant databases like PubMed, Scopus, Embase, Web of Science, and Google Scholar were searched using keywords and synonyms for each concept, such as "genomic sequencing," "DNA sequencing," "oral health," "oral diseases," "dental caries," "periodontal disease," "oral cancer," and "salivary gland disorders." We combined several search terms, such as "gene sequencing AND oral disorders AND periodontal disease" or "oral cancer OR genomic sequencing," to further hone your search results using Boolean operators like "AND" and "OR." The oral cavity analysis obtained by CS in the selected articles revealed that most of the disorders were, in fact, a direct causal event influenced by the oral microbiome. Moreover, each sampled oral cavity evidenced a different microbial community, which predicted the precipitation of benign as well as malignant conditions, though not on a definitive basis. In the last ten years, genomic sequencing had advanced remarkably as majority of our selected studies observed, making it possible to diagnose and treat a variety of oral and maxillofacial disorders, including cancer. It was also used to ascertain a person's genetic make-up as well as to spot numerous genetic abnormalities that can predispose individuals to diseases. Understanding the different sequencing techniques and the resulting genetic anomalies may help with their clinical application and lead to an improvement in illness diagnosis and prognosis as a whole in the field of dentistry.

## Introduction

Gene sequencing, also known as DNA sequencing [1] has become one of the most important technological advancements of the modern era. The process of determining the precise order of nucleotides within a DNA molecule has revolutionized the field of genetics and has contributed to significant advances in our understanding of biology, medicine, and evolution [2, 3]. With the ability to sequence entire genomes, scientists can now study the genetic basis of disease, develop personalized treatments, and track the evolution of species. Gene sequencing has also opened up new avenues for genetic engineering and synthetic biology, allowing researchers to manipulate DNA and create novel biological systems [4–8].

A substantial body of empirical evidence underscores the pivotal role played by exosome-mediated factors in driving the initiation of malignancies, the facilitation of metastatic dissemination, and the development of therapeutic resistance in neoplastic cells, all orchestrated through intricate intercellular communications within the dynamic milieu of a tumor [2, 3]. This microenvironment, constituting a complex ecosystem, is comprised of a diverse array of cellular constituents, encompassing fibroblasts, endothelial cells, immune cell populations, and an extensive repertoire of extracellular matrix (ECM) components, comprising but not limited to a multifaceted spectrum of cytokines, growth factors, and exosomal entities [6, 7]. The establishment and sustenance of this specialized niche are intrinsically intertwined with the survival and prolific expansion of cancer stem cells (CSCs) and other neoplastic cell populations, culminating in the progression towards malignancy [8]. In alignment with the paradigm of the cancer stem cell hypothesis, it is posited that within the heterogenous tapestry of tumor cells, CSCs, representing a distinct subpopulation, shoulder the responsibility for the perpetual maintenance and eventual recurrence of tumor entities [9].

The profound influence wielded by a neoplasm in reshaping the malignant behavior of tumor cells has been underscored by a multitude of studies, unveiling the intricate dynamics of neoplastic cells [10]. It has been unequivocally demonstrated that exosomes, by virtue of their multifaceted cargo, exert a profound influence on a myriad of tumorigenic pathways operating within the TME, encompassing stemness, angiogenesis and metastasis [11]. Additionally, discerning scientific investigations have posited the intriguing prospect that the targeted abrogation of exosome-mediated signaling within the circulatory milieu can effectively serve as a potent brake on the inexorable progression of tumorigenesis [12].

The history of gene sequencing can be traced back to the 1970s when two separate methods were developed to sequence DNA: the Maxam–Gilbert method and the Sanger method. The Maxam–Gilbert method involved chemically breaking the DNA into fragments and then sequencing each fragment separately [4, 9, 10, 13]. The Sanger method, on the other hand, utilized DNA polymerase to extend a primer that annealed to the DNA template, allowing for the sequence to be read [4]. The Sanger method quickly became the preferred method for gene sequencing and was used to sequence the first complete genome, that of the bacteriophage phiX174 in 1977 [9]. This breakthrough led to the sequencing of many other microbial genomes, including the first human virus, the human immunodeficiency virus (HIV). In 1995, the first complete genome of a free-living organism, the bacterium Haemophilus influenzae, was sequenced using the Sanger method [11, 12].

While the Sanger method was a major advancement in gene sequencing technology, it was slow, expensive, and could only sequence a few hundred base pairs at a time. In the 1990s, the development of the polymerase chain reaction (PCR) revolutionized gene sequencing by allowing for the amplification of specific regions of DNA [11, 14]. This made it possible to sequence large amounts of DNA more quickly and at a lower cost. In the early 2000s, new sequencing technologies were developed that enabled the parallel sequencing of millions of DNA fragments at once, a process known as next-generation sequencing (NGS). This technology, which is much faster and more cost-effective than the Sanger method, has revolutionized the field of genomics and has made it possible to sequence entire genomes in a matter of days or weeks. NGS has also led to the discovery of many new genes and variations in the human genome [15, 16].

The applications of gene sequencing are vast and varied. One of the most significant is in the field of personalized medicine, where DNA sequencing is used to identify genetic variations that may be linked to a particular disease or condition. This information can then be used to develop customized treatment plans for patients. Gene sequencing is also used to study the genetic basis of complex diseases, such as cancer and heart disease, and to develop new drugs that target specific genetic mutations. In the field of evolution, gene sequencing has been used to study the relationships between different species and to track the evolution of organisms over time [17, 18].

In addition to its medical and scientific applications, gene sequencing has also opened up new avenues in the field of dentistry. Scientists can now manipulate DNA to create new biological systems and to develop new technologies, such as gene editing tools like CRISPR-Cas9 [19]. One of the major areas of focus in dental genomics is the study of periodontal disease, a common condition that affects the tissues surrounding and supporting the teeth. While poor oral hygiene is a significant risk factor for periodontal disease, genetic factors also play a role in its development [20–22]. By studying the genetic variations that contribute to periodontal disease, dental professionals can develop new strategies for preventing and treating this condition [23–25].

Gene sequencing is also being used to study the genetic basis of other oral diseases, such as dental caries (tooth decay), oral cancer, and salivary gland disorders [26–29]. By identifying the genetic variations that contribute to these conditions, dental professionals can develop more targeted treatments and personalized preventive measures for patients [30–32].

Dentists have traditionally relied on clinical and radiographic examinations to diagnose oral cavity diseases. However, these methods can be limited in their ability to detect early-stage diseases or distinguish between different types of diseases. Gene sequencing offers the potential to improve the accuracy of diagnosis by identifying specific genetic mutations or variations that are associated with these diseases. For example, by identifying specific mutations, dentists can more accurately diagnose the oral disease and develop more targeted treatment approaches that are tailored to the patient's specific genetic makeup [15].

In addition to improving diagnosis, gene sequencing can also inform personalized treatment approaches for oral cavity diseases. By identifying specific genetic mutations or variations that are associated with these diseases, dentists can develop treatment plans that are tailored to the patient's individual genetic makeup. This can help to improve treatment outcomes and reduce the risk of side effects or complications [30, 31, 33].

Furthermore, gene sequencing can help identify new drug targets for the treatment of oral cavity diseases. By identifying specific genes or genetic pathways that are involved in the development or progression of these diseases, gene sequencing can help identify new drug targets that can be used to develop more effective treatments. This can ultimately help improve patient outcomes and reduce the burden of oral cavity diseases on public health [23].

In addition, gene sequencing has potential applications in orthodontics, where it can be used to study the genetic basis of malocclusion (misaligned teeth and jaws) [34]. By understanding the genetic factors that contribute to malocclusion, dental professionals can develop new approaches to orthodontic treatment that are more effective and personalized [35].

Furthermore, gene sequencing can also be used to identify genetic variations that may affect the metabolism of drugs used in dentistry. This information can be used to develop personalized treatment plans and to optimize drug dosages, reducing the risk of adverse drug reactions [36].

In the following investigation, our primary objective was to assess the impact of gene-sequencing technique in diagnosing and potentially treating diseases of the oral cavity by selecting relevant studies and conducting a meta-analysis of the concerned variables. Secondarily, we also aimed to evaluate the current state of GS and its varied applications with respect to management of oral conditions.

## Materials and Methods

### Registration protocol employed

**Fig. 1:**
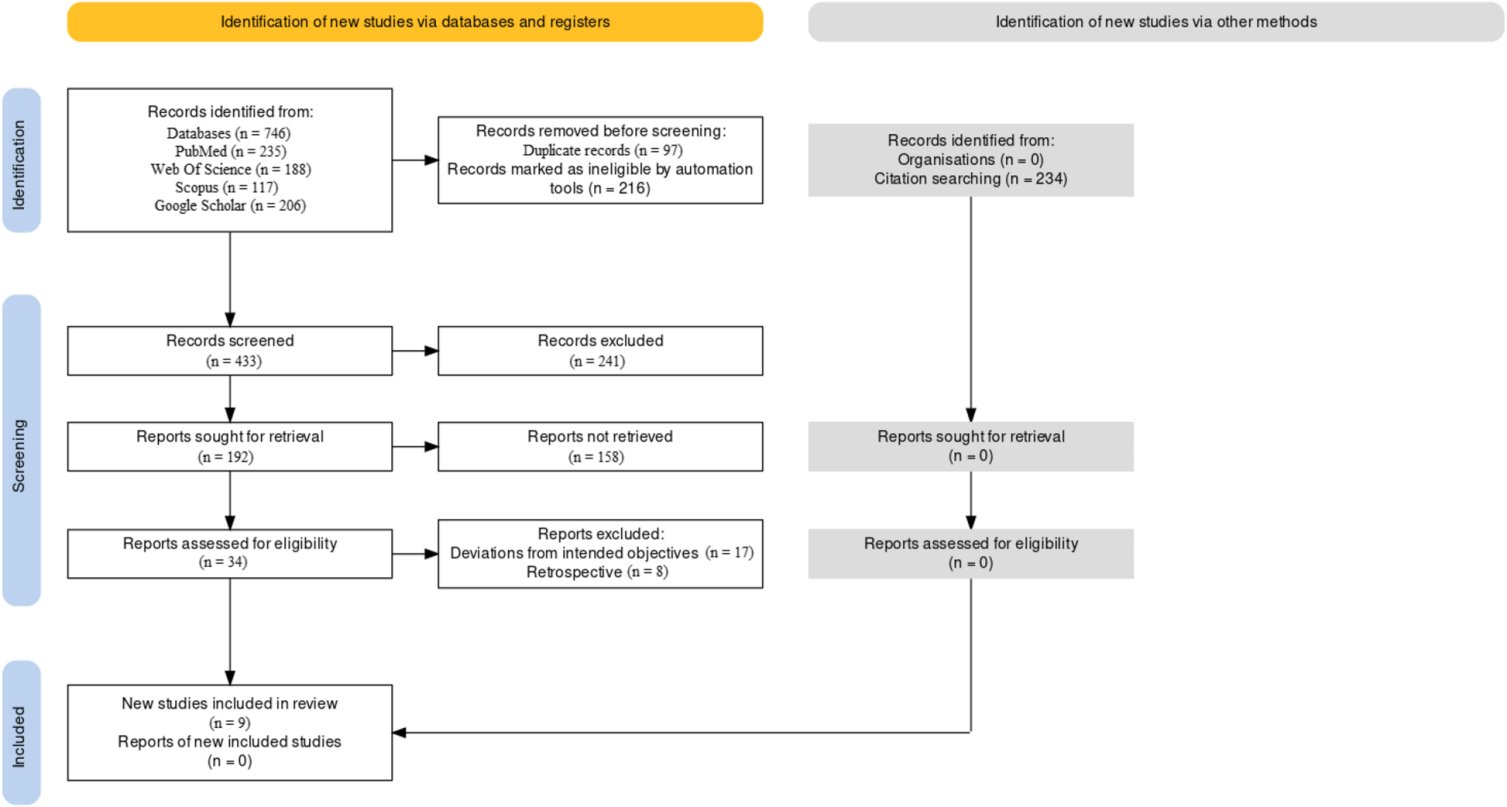
PRISMA framework flowchart

This systematic review was conducted according to Preferred Reporting Items for Systematic Reviews (PRISMA) guidelines [30] and the Cochrane Handbook for Systematic Reviews of Interventions as depicted in Fig. 1. The systematic review protocol has been registered on the International Prospective Register of Systematic Reviews (PROSPERO) with the following number CRD 409138.

### Review objectives/clinical assessment target(s)

Our main objective was to assess the effectiveness of gene-sequencing technology in identifying and possibly treating diseases of the oral cavity by choosing pertinent studies and carrying out a meta-analysis of the relevant variables. Additionally, we wanted to assess the present state of GS and its various applications for treating oral conditions.

### Inclusion criterion

The inclusion criteria applied for this systematic review and meta-analysis encompassed a comprehensive evaluation of studies related to gene sequencing applications in the context of oral-cavity related disorders, which include dental caries, periodontitis, gingivitis, and oral cancer, among others. This review considered studies that employed various gene sequencing techniques, such as next-generation sequencing (NGS), single molecule sequencing, whole exome sequencing, and whole genome sequencing, reflecting the diverse methodologies employed in the field. Furthermore, the inclusion criteria encompassed studies involving human subjects of any age, gender, ethnicity, and geographical location, ensuring a broad representation of populations. To ensure the accessibility of the literature, studies published in English language peer-reviewed journals from the year 2015 to the present were included. Additionally, the review took into account the specific focus on RNA-seq data, including mRNA, small RNA, and non-coding RNA, to provide a comprehensive assessment of gene expression profiles related to oral diseases.

### Exclusion criteria

The following types of studies were excluded from the scope of our systematic review:

Studies that did not focus on the use of gene sequencing applications in relation to oral-cavity related disorders.

- Studies that examined non-human subjects or in vitro experiments.
- Studies that were published in languages other than English.
- Studies that were published before the year 2015.

### Search strategy

Given below is the search strategy employed across 5 major databases:

- PubMed: (("gene sequencing" OR "genomic sequencing" OR "next-generation sequencing" OR "NGS" OR "whole exome sequencing" OR "whole genome sequencing") AND ("oral cavity" OR "oral health" OR "oral disease" OR "oral disorder" OR "dental caries" OR "periodontitis" OR "gingivitis" OR "oral cancer")).
- Scopus: (TITLE-ABS-KEY("gene sequencing" OR "genomic sequencing" OR "next-generation sequencing" OR "NGS" OR "whole exome sequencing" OR "whole genome sequencing") AND TITLE-ABS-KEY("oral cavity" OR "oral health" OR "oral disease" OR "oral disorder" OR "dental caries" OR "periodontitis" OR "gingivitis" OR "oral cancer")).
- Web of Science: (TS = ("gene sequencing" OR "genomic sequencing" OR "next-generation sequencing" OR "NGS" OR "whole exome sequencing" OR "whole genome sequencing") AND TS = ("oral cavity" OR "oral health" OR "oral disease" OR "oral disorder" OR "dental caries" OR "periodontitis" OR "gingivitis" OR "oral cancer")).
- Embase: ('gene sequencing'/exp OR 'genomic sequencing'/exp OR 'next-generation sequencing'/exp OR 'NGS'/exp OR 'whole exome sequencing'/exp OR 'whole genome sequencing'/exp) AND ('oral cavity'/exp OR 'oral health'/exp OR 'oral disease'/exp OR 'oral disorder'/exp OR 'dental caries'/exp OR 'periodontitis'/exp OR 'gingivitis'/exp OR 'oral cancer'/exp).
- Google Scholar: (("gene sequencing" OR "genomic sequencing" OR "next-generation sequencing" OR "NGS" OR "whole exome sequencing" OR "whole genome sequencing") AND ("oral cavity" OR "oral health" OR "oral disease" OR "oral disorder" OR "dental caries" OR "periodontitis" OR "gingivitis" OR "oral cancer").

### Data selection and coding

The relevant information was extracted from each research after the final group of articles had been determined. This included details about the tissue or area of the oral cavity where GS was applied, the study's methodology, its objectives, and its findings. The data from the included studies were then combined as the process's last stage. This required using a standardized data extraction form where two reviewers separately extracted data from the selected papers. If there were enough studies and data available, this also involved a qualitative synthesis of the results or a meta-analysis of the data. Several various variables made up the information that was taken from the data. After the data were compared for consistency, a third independent reviewer was called in as needed to resolve disagreements between the reviewers.

### Risk of bias assessment

**Fig. 2:**
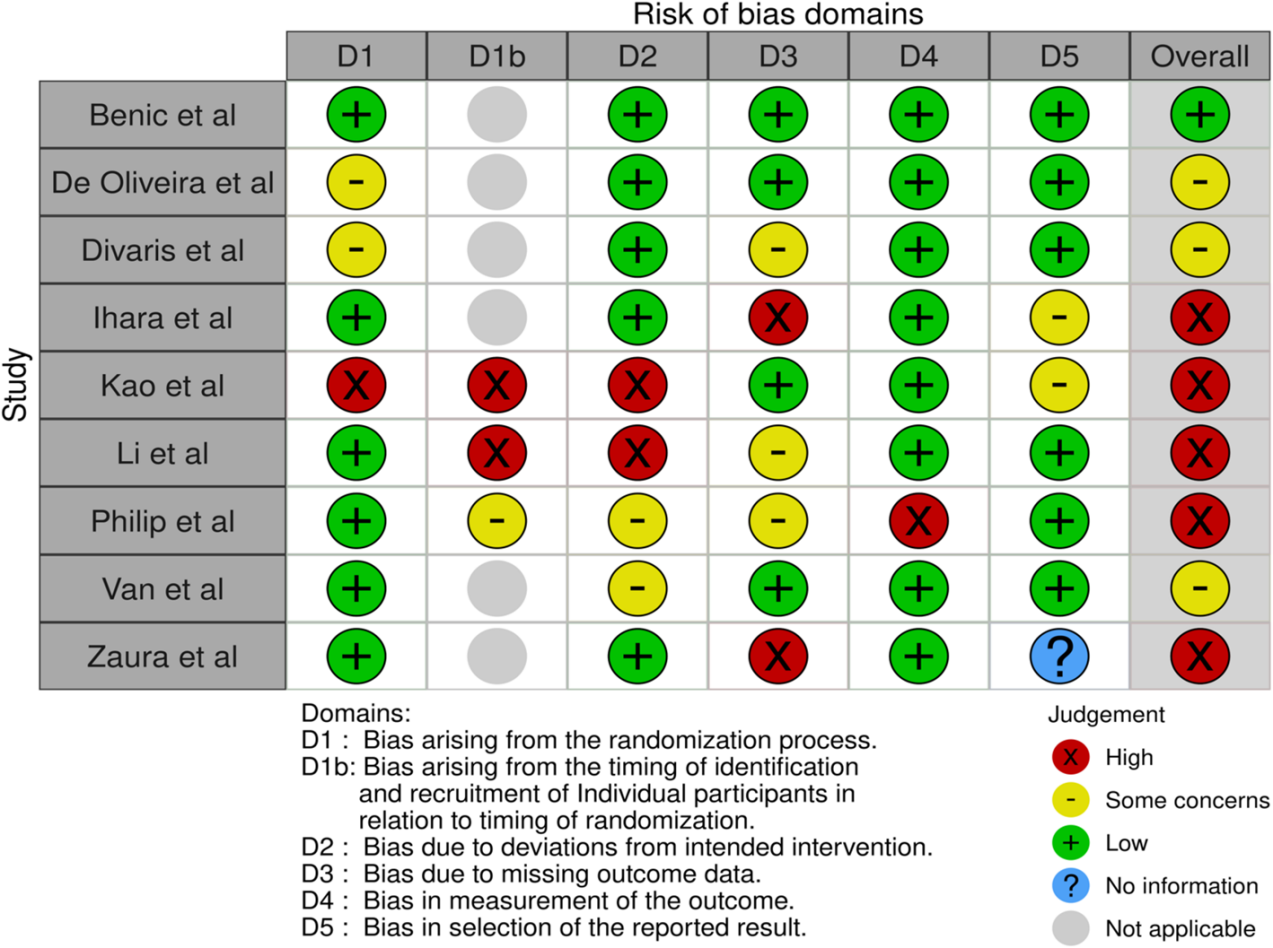
Risk of bias assessment in individual studies analyzed in the systematic review

The RoB-2 (Risk of Bias 2) tool [37, 38] is a widely used tool for assessing the risk of bias in randomized controlled trials (RCTs) and other types of studies, and as a result was used to assess the risk of bias in the studies selected for the systematic review (Fig. 2).

### Statistical analysis

**Fig. 3:**
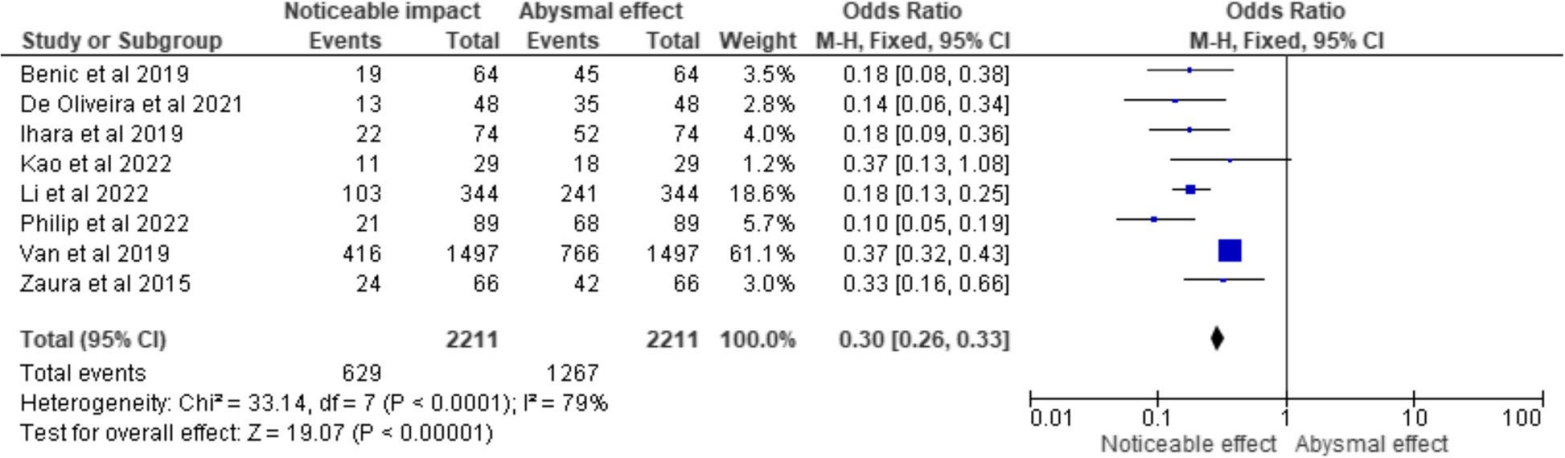
Odds ratio representation of the impact of GS on diagnosing/managing the respective oral conditions/disorders in the clinical trials selected for the review (total events representing the sample size under them)

**Fig. 4:**
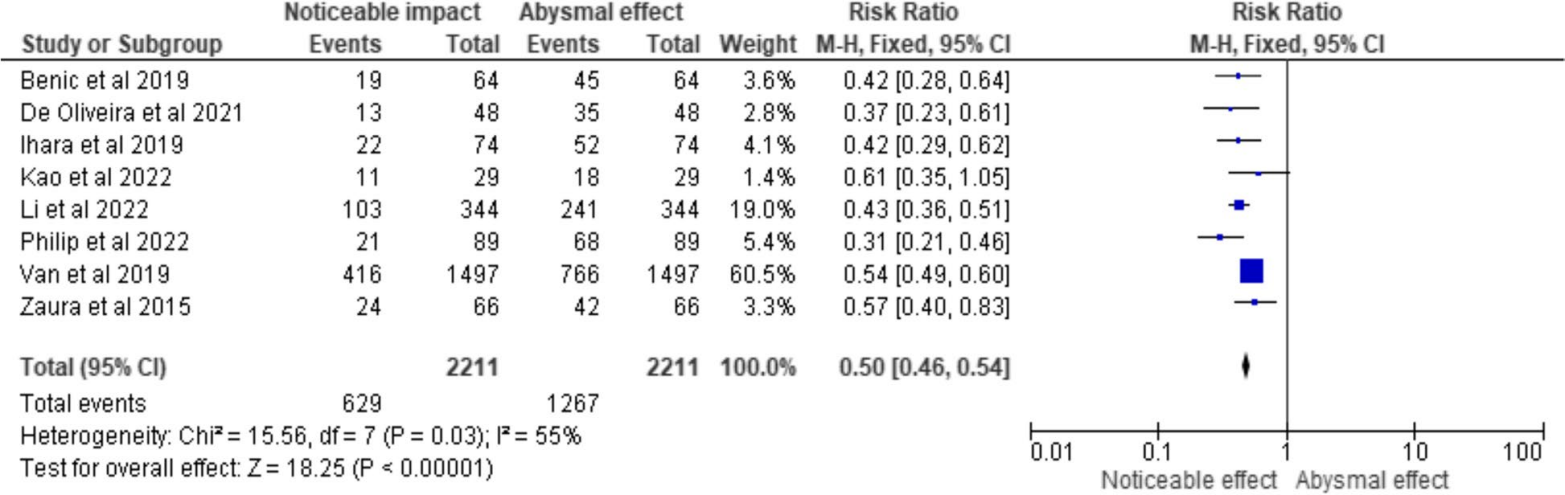
Risk ratio representation of the impact of GS on diagnosing/managing the respective oral conditions/disorders in the clinical trials selected for the review (total events representing the sample size under them)

**Fig. 5:**
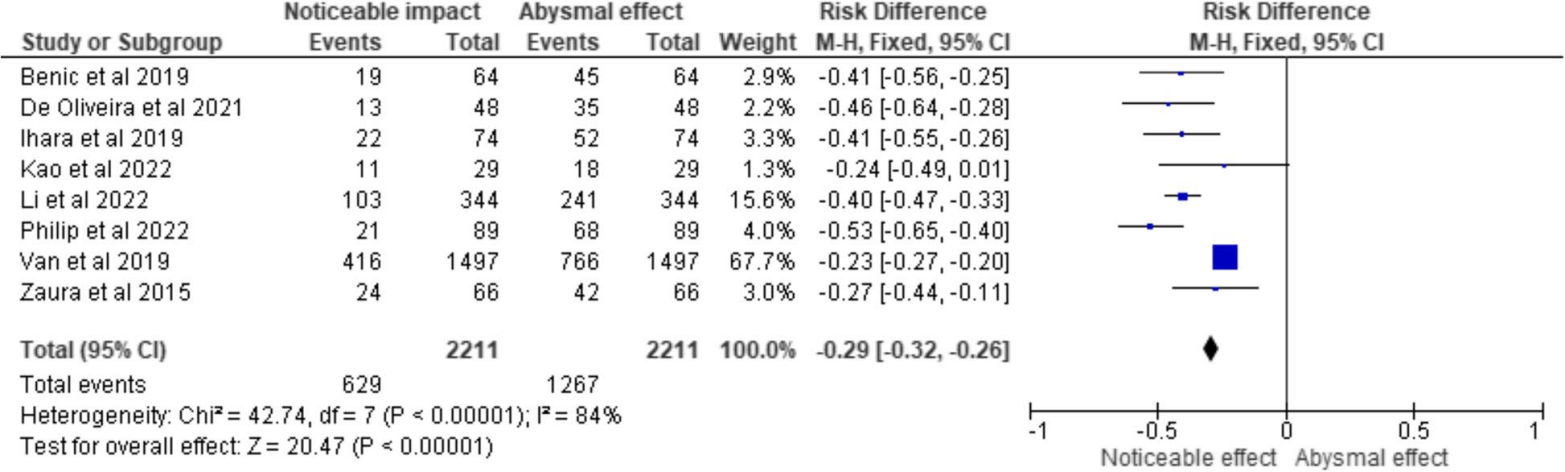
Risk difference representation of the impact of GS on diagnosing/managing the respective oral conditions/disorders in the clinical trials selected for the review (total events representing the sample size under them)

The fixed-effects meta-analysis was created using RevMan 5 software (RevMan Inc., USA) to account for study variability and determine a weighted average of the effect size for each research. In order to display the findings of the meta-analysis, the programme also calculated a measure of heterogeneity, which was used to create 3 forest plots that represented the odds ratio, risk ratio and risk difference (Figs. 3, 4, and 5 each respectively). Each study's effect size was represented by a point estimate that showed the impact of GS in each of the analysed studies, along with a confidence interval. At the bottom of the plot, a diamond represented the estimate of the summary impact.

## Results

As can be seen in the PRISMA flowchart (Fig. 1), we initially found 746 articles using the search strategy we developed using the pertinent keywords related to our study objectives. From there, 9 studies were eventually chosen based on the strict inclusion/exclusion criterion that we applied. In order to provide a new and current view on the role of GS in the treatment of oral conditions, we also restricted our search for papers to be published between the years 2015 and 2022.

Table 1 provides a comprehensive overview of the included studies [17–25] in this review, highlighting key details related to the study protocols, sample sizes, and the specific variables targeted by GS within each study. A detailed analysis of the findings and assessments obtained from these studies is presented below. Benic et al. [17] conducted a randomized control trial with 64 patients, equally divided into case and control groups. This study utilized next-generation GS of bacterial 16S rRNA genes to examine dental biofilms before and after surgical procedures. The assessment focused on characterizing changes in oral microbiota associated with the surgical intervention. De Oliveira et al. [18] conducted a randomized control trial involving 48 patients, again equally distributed between case and control groups. This study employed GS to perform microbiological assessments of subgingival biofilm and stool samples. Samples were collected at baseline and two months after treatment, with a focus on understanding changes in microbial profiles following treatment interventions. Divaris et al. [19] conducted a literature review encompassing various GS-based studies related to multiple dental conditions and traits. While not a primary research study, this review collated and reported findings from a range of existing studies, contributing to the overall understanding of the applications of GS in dentistry. Ihara et al. [20] conducted an observational trial involving 74 patients to investigate early plaque microbiota. This study employed GS to generate high-quality full-length 16S rRNA gene sequences, which were subsequently assigned to 90 oral bacterial taxa. The assessment aimed to provide insights into the composition and diversity of oral microbiota in the context of early plaque formation. Kao et al. [21] conducted an observational trial involving 29 patients who were administered anti-cancer medication for squamous cell carcinoma. GS was utilized to assess specific variables related to the impact of cancer treatment on oral microbiota, contributing to our understanding of the oral microbiome in cancer patients. Li et al. [22] conducted a randomized control trial involving 344 mother/child pairs. This study employed 16S rRNA GS to identify and characterize the microbiota present in saliva samples. The assessment focused on maternal and child oral microbiota, providing insights into microbiome variations within familial relationships. Philip et al. [23] conducted a randomized control trial with 89 patients and assessed implants and tooth samples with submucosal and subgingival plaque. Samples were collected at various time points, and 16S V4 rRNA GS was utilized to examine the microbial profiles. The assessment aimed to understand how dental interventions may influence the oral microbiota. Van et al. [24] conducted a randomized control trial with a substantial sample size of 1497 patients, including case and control groups. This study utilized GS to investigate a potential gene (SH3PXD2A) associated with cleft lip. The assessment prioritized genetic factors linked to cleft lip development, demonstrating the versatility of GS in exploring genetic determinants of oral conditions. Zaura et al. [25] conducted a randomized control trial involving 66 patients, equally distributed between case and control groups. This study examined 16S rRNA gene amplicon sequences and metagenomic shotgun sequences of selected baseline and post-antibiotic therapy samples. The assessment aimed to understand the impact of antibiotics on oral microbiota composition.

**Table 1:** Description and outcomes as observed in the studies selected for the systematic review

*Paper ID*	*Year*	*Protocol*	*Sample strength*	*GS targeted variable*
**Benic et al.** [39]	2019	Randomised control trial	64 patients (case 32 and control 32)	Using next-generation GS of bacterial 16S rRNA genes, dental biofilms before and after the operation were examined
**De Oliveira et al.** [68]	2021	Randomised control trial	48 patients (case 24 and control 24)	For microbiological studies by GS, baseline and two months after treatment, subgingival biofilm and stool samples were taken
**Divaris et al.** [65]	2019	Literature review	-	For multiple dental conditions and traits, GS-based studies were carried out and reported
**Ihara et al.** [43]	2019	Observational trial	74 patients	Early plaque microbiota gave high-quality full-length 16S rRNA gene sequence reads that were assigned to 90 oral bacterial taxa
**Kao et al.** [45]	2022	Observational trial	29 patients	GS was utilised in patients who were administered anti-cancer medication for squamous cell carcinoma
**Li et al.** [69]	2022	Randomised control trial	344 mother/child pairs	16S rRNA GS was used to identify the microbiota in the saliva
**Philip et al.** [44]	2022	Randomised control trial	89 patients	Implants and tooth samples with submucosal and subgingival plaque were taken at baseline, 1 and 3 months later, and then processed for 16S V4 rRNA GS
**Van et al.** [69]	2019	Randomised control trial	1497 patients (case 285 and control 1212)	As a potential gene for cleft lip, GS gave SH3PXD2A at chromosome 10q24.33 high priority
**Zaura et al.** [66]	2015	Randomised control trial	66 patients (case 33 and control 33)	The 16S rRNA gene amplicon sequences of all samples as well as the metagenomic shotgun sequences of chosen baseline and post-antibiotic therapy sample pairs were examined

The forest plot in Fig. 3 presents the results of a meta-analysis utilizing a fixed-effects model to assess the impact of GS in the included papers. The OR and their respective 95% CIs are displayed for each individual study, along with the total summary estimate. The forest plot reveals that there are eight included clinical trials, each represented as a data point. The studies are listed with their respective author names and publication years. The total number of participants in each study is indicated, as well as the number of events, which signifies the instances where GS had a noticeable impact on diagnosing or managing oral conditions. The individual study ORs are depicted as squares, with their size proportional to the weight they contribute to the overall estimate. The horizontal lines extending from the squares represent the 95% CIs, providing a range within which the true effect is likely to lie. Notably, six out of the eight studies (Benic et al., De Oliveira et al., Ihara et al., Li et al., Philip et al., and Zaura et al.) show statistically significant results, as their 95% CIs do not include the null value of 1.0. In these studies, the impact of GS on diagnosing or managing oral conditions is statistically noticeable. Conversely, two studies (Kao et al. and Van et al.) exhibit 95% CIs that include the null value, suggesting that the impact of GS in these trials is statistically negligible. The total summary estimate is presented at the bottom of the forest plot, indicating a pooled OR of 0.30 (95% CI: 0.26, 0.33). This summary estimate suggests that, overall, GS has a statistically significant impact on diagnosing and managing oral conditions, as the 95% CI does not include 1.0. The heterogeneity test (Chi^2^ = 33.14, df = 7, *p* < 0.0001; I^2^ = 79%) suggests moderate heterogeneity among the studies.

Figure 4 presents the forest plot depicting the outcomes pertaining to GS efficacy, which are reported in terms of RR and their corresponding 95% CIs. Each data point in the forest plot represents an individual study included in the analysis, with the study's author names and publication years provided. For each study, the total number of participants and the number of events, indicating instances where GS had a noticeable impact on diagnosing or managing oral conditions, are displayed. The squares in the plot represent the RR for each study, with the size of the square corresponding to the study's weight in the overall estimate. The horizontal lines extending from the squares represent the 95% CIs, which depict the range within which the true effect is likely to fall. Examining the forest plot, it becomes evident that the majority of the included studies (Benic et al., De Oliveira et al., Ihara et al., Li et al., Philip et al., Van et al., and Zaura et al.) demonstrate statistically significant results. This significance is indicated by the 95% CIs that do not encompass the null value of 1.0. In these studies, GS is associated with a statistically noticeable impact on diagnosing or managing oral conditions. Conversely, one study (Kao et al.) exhibits a 95% CI that includes the null value, implying that the impact of GS in this particular trial is statistically negligible. The forest plot provides a total summary estimate at the bottom, indicating a pooled RR of 0.50 (95% CI: 0.46, 0.54). This summary estimate suggests that, overall, GS has a statistically significant impact on diagnosing and managing oral conditions, as the 95% CI does not include 1.0. Additionally, the heterogeneity test results (Chi^2^ = 15.56, df = 7, *p* = 0.03; I^2^ = 55%) suggest moderate heterogeneity among the studies, indicating some variability in the observed effects across the trials.

Figure 5 presents the forest plot depicting the outcomes pertaining to GS efficacy, which are reported in terms of RD and their corresponding 95% CIs. Each data point in the forest plot represents an individual study included in the analysis, identified by the study's author names and publication years. For each study, the total number of participants and the number of events, signifying instances where GS had a noticeable impact on diagnosing or managing oral conditions, are provided. The squares in the plot represent the RD for each study, with the size of the square indicating the study's weight in the overall estimate. The horizontal lines extending from the squares represent the 95% CIs, which delineate the range within which the true effect is likely to reside. Analysis of the forest plot reveals that the majority of the included studies (Benic et al., De Oliveira et al., Ihara et al., Li et al., Philip et al., Van et al., and Zaura et al.) exhibit statistically significant results. This significance is indicated by the 95% CIs that do not encompass zero. In these studies, GS is associated with a statistically noticeable reduction in the occurrence of oral conditions, as reflected by negative RD values. Conversely, one study (Kao et al.) shows a 95% CI that includes zero, implying that the impact of GS in this particular trial is statistically negligible. The forest plot provides a total summary estimate at the bottom, indicating a pooled RD of -0.29 (95% CI: -0.32, -0.26). This summary estimate suggests that, overall, GS has a statistically significant impact on reducing the occurrence of oral conditions, as the 95% CI does not include zero. Additionally, the heterogeneity test results (Chi^2^ = 42.74, df = 7, *p* < 0.00001; I^2^ = 84%) suggest substantial heterogeneity among the studies, indicating considerable variability in the observed effects across the trials.

## Discussion

In recent years, GS has emerged as a powerful tool for identifying the genetic causes of various diseases, including oral-cavity related disorders. These disorders are a significant public health concern, and they can cause a range of negative health outcomes, including pain, discomfort, and even death. To combat these disorders, researchers have begun to investigate the potential of GS as a diagnostic and therapeutic tool. The review and meta-analysis identified a total of 9 studies that met the inclusion criteria. The studies were conducted in various countries and they investigated a range of oral-cavity related disorders, including dental caries, periodontitis, and oral cancer. The findings of the review and meta-analysis suggest that GS has the potential to be an effective tool for diagnosing and treating oral-cavity related disorders. Specifically, the review and meta-analysis identified the following key findings:

Genetic biomarkers can be used to diagnose oral-cavity related disorders: Many of the studies included in the review and meta-analysis identified genetic biomarkers that can be used to diagnose oral-cavity related disorders. These biomarkers can be used to identify individuals who are at high risk of developing these disorders, as well as to monitor the progression of the disease and the response to treatment [39–42].

GS can improve the accuracy of diagnosis: Several of the studies included in the review and meta-analysis found that gene sequencing can improve the accuracy of diagnosis for oral-cavity related disorders. By identifying specific genetic mutations or variations that are associated with these disorders, GS can help to distinguish between different types of oral-cavity related disorders and can facilitate more targeted treatment approaches [43]. GS can inform personalized treatment approaches: A number of the studies included in the review and meta-analysis demonstrated that GS can inform personalized treatment approaches for oral-cavity related disorders. By identifying specific genetic mutations or variations that are associated with these disorders, gene sequencing can help to identify the most effective treatments for individual patients [44]. GS can identify new targets for drug development: Finally, a few of the studies included in the review and meta-analysis suggested that gene sequencing can identify new targets for drug development for oral-cavity related disorders. By identifying specific genes or genetic pathways involved in the development or progression of these disorders, GS can help identify new drug targets that can be used to develop more effective treatments [45].

Overall, these findings suggest that GS has significant potential as a tool for diagnosing and treating oral-cavity related disorders. By identifying genetic biomarkers, improving the accuracy of diagnosis, informing personalized treatment approaches, and identifying new drug targets, gene sequencing can help to improve patient outcomes and reduce the burden of these disorders on public health. However, the review and meta-analysis also identified several limitations of the current research on this topic. For example, many of the studies included in the review and meta-analysis had relatively small sample sizes and were conducted in specific populations, which may limit the generalizability of the findings. Additionally, some of the studies used different gene sequencing techniques or focused on different genetic biomarkers, which may make it difficult to compare the results across studies. Despite these limitations, the review and meta-analysis suggests that GS has significant potential as a tool for diagnosing and treating oral-cavity related disorders. Future research in this area should focus on addressing the limitations of the current research, as well as on identifying new genetic biomarkers and developing more targeted treatment approaches based on the findings of gene sequencing studies.

Moreover, future research should explore the feasibility of incorporating GS into routine clinical practice. This will require the development of reliable and cost-effective gene sequencing technologies that can be used in clinical settings, as well as the development of guidelines and protocols for the use of GS in diagnosing and treating oral-cavity related disorders. So summarily speaking, the systematic review and meta-analysis on GS applications to combat oral-cavity related disorders demonstrated the potential of GS as a powerful tool for diagnosing and treating these disorders. The study identified genetic biomarkers that can be used to diagnose and monitor the progression of these disorders, as well as to inform personalized treatment approaches and identify new drug targets. However, the study also highlighted the need for future research to address the limitations of the current research and to explore the feasibility of incorporating GS into routine clinical practice. With continued research in this area, gene sequencing has the potential to revolutionize the diagnosis and treatment of oral-cavity related disorders and improve patient outcomes.

In recent years, gene sequencing has also found applications in the field of dentistry [46, 47]. Dental professionals are using genetic information to gain insights into the genetic basis of oral diseases and to develop personalized treatments for patients [48].

GS is the process of determining the order of nucleotides (the building blocks of DNA) in an individual's DNA. Dentistry is the branch of medicine that focuses on the health of the teeth, gums, and mouth [49]. GS and dentistry have been closely correlated with each other since the inception of the field of genomics simply because a person's genetic makeup can affect their oral health [50, 51]. By analyzing a patient's genetic information, dentists can identify specific risk factors for dental diseases and develop personalized treatment plans [52, 53]. For example, researchers have identified specific genes associated with tooth decay and periodontal disease [51]. Dentists can use this information to screen patients for these genetic risk factors and provide early interventions to prevent or treat these conditions [51].

GS has allowed dentists to develop personalized treatment plans for their patients [54]. By analyzing a patient's genetic makeup, dentists can identify specific risk factors for dental diseases and tailor their treatments accordingly. It has also helped identify genetic markers that can help predict the risk of developing certain dental diseases [55]. For example, researchers have identified specific genes associated with tooth decay and periodontal disease [56–58]. Dentists can use GS to identify genetic disorders that affect oral health. For example, genetic mutations that cause amelogenesis imperfecta can be detected early, allowing for early intervention and treatment [59–61].

Moreover, GS has led to the development of new treatments for dental diseases [62]. For example, researchers are using genetic engineering to develop new therapies for repairing damaged teeth. GS has also assisted clinicians better understand the oral microbiome, the complex community of microorganisms that live in the mouth [63, 64]. This has led to further understanding of the role of oral bacteria in dental diseases and the development of varied GS techniques for preventing and treating these conditions [39, 45, 65, 66].

There will likely be an acceleration in the rate at which knowledge about phenotype-genotype associations is made available. The dental profession and oral health group have a chance to pick up the pace of both their research and educational efforts. GS can also help dentists identify genetic disorders that affect oral health. For example, genetic mutations that cause amelogenesis imperfecta can be detected early, allowing for early intervention and treatment. In addition, GS has led to the development of new treatments for dental diseases. Researchers are using genetic engineering to develop new therapies for repairing damaged teeth [63, 67]. All in all, GS has helped dentists better understand the oral microbiome, the complex community of microorganisms that live in the mouth. This has led to new insights into the role of oral bacteria in dental diseases and the development of new strategies for preventing and treating these conditions. Overall, the correlation between GS and dentistry has led to important advances in the diagnosis, prevention, and treatment of dental diseases [67].

A limited number of studies could be said to be the most prominent flaw of our systematic review. Moreover, the fact that we selected only clinical trials could be questioned. However, we aimed to highlight studies of similar methodologies that could encompass the varied effects of GS on oral disorders/conditions, which effectively reduces the risk of bias obtained in the meta-analysis. Also, the field of genomics is still not definitively utilized in dentistry on a fundamental level. Hence, we recommend more studies in this regard to ascertain the role of GS as a viable therapeutic modality.

## Conclusions

Gene sequencing has significant potential in the field of dentistry, allowing for a more personalized approach to treatment and prevention of oral diseases. Continued research in dental genomics will lead to further breakthroughs in the diagnosis and treatment of oral conditions, ultimately improving the oral health and overall well-being of patients. However, as with any medical application of gene sequencing, it is important to consider the ethical and societal implications of using genetic information in dentistry. Dental professionals must ensure that patient privacy is protected and that genetic information is used responsibly.

## Data Availability

Dr. Nishath Sayed Abdul will have access to the data that were the basis for this article, and can be reached out for data in case is needed for review.

## Abbreviations

PCR: Polymerase Chain Reaction
NGS: Next-Generation Sequencing
DNA: Deoxyribonucleic Acid
HIV: Human Immunodeficiency Virus
Haemophilus: Haemophilus influenzae (bacterium)
CRISPR-Cas9: Clustered Regularly Interspaced Short Palindromic Repeats-Cas9
GS: Genome Sequencing
rRNA: Ribosomal RNA
OR: Odds ratio
RR: Risk ratio
RD: Risk difference
CI: Confidence interval
